# Assessment of Pulmonary Function Tests in COVID-19 Convalescents Six Months after Infection

**DOI:** 10.3390/jcm11237052

**Published:** 2022-11-29

**Authors:** Katarzyna Guziejko, Anna Moniuszko-Malinowska, Piotr Czupryna, Marlena Dubatówka, Magda Łapińska, Andrzej Raczkowski, Paweł Sowa, Łukasz Kiszkiel, Łukasz Minarowski, Marcin Moniuszko, Monika Groth, Karol A. Kaminski

**Affiliations:** 1 2nd Department of Lung Diseases and Tuberculosis, Medical University of Bialystok, Zurawia 14, 15-540 Bialystok, Poland; lukasz.minarowski@gmail.com; 2 Department of Infectious Diseases and Neuroinfections, Medical University of Bialystok, Zurawia 14, 15-540 Bialystok, Poland; annamoniuszko@op.pl (A.M.-M.); avalon-5@wp.pl (P.C.); mkrol94@gmail.com (M.G.); 3 Department of Population Medicine and Lifestyle Diseases Prevention, Medical University of Bialystok, ul. J. Kilinskiego 1, 15-259 Bialystok, Poland; marlena.paniczko@umb.edu.pl (M.D.); magda.lapinska@umb.edu.pl (M.Ł.); andrzej.raczkowski@umb.edu.pl (A.R.); mailtosowa@gmail.com (P.S.); fizklin@wp.pl (K.A.K.); 4 Society and Cognition Unit, University of Bialystok, 15-403 Bialystok, Poland; lukaszkiszkiel@gmail.com; 5 Department of Regenerative Medicine and Immune Regulation, Medical University of Bialystok, Waszyngtona 13, 15-269 Bialystok, Poland; marcin.moniuszko@umb.edu.pl; 6 Department of Allergology and Internal Medicine, Medical University of Bialystok, M. Sklodowskiej-Curie 24A, 15-276 Bialystok, Poland; 7 Department of Cardiology, Medical University of Bialystok, M. Sklodowskiej-Curie 24A, 15-276 Bialystok, Poland

**Keywords:** COVID-19, pulmonary function test, shortness of breath, cough, recovery

## Abstract

Background: The aim of the study was to investigate the impact of COVID-19 on the pulmonary function tests (PFT) in COVID-19 convalescents six months after recovery. Additionally, the research question was whether PFT should be performed routinely in post-COVID-19 patients. Methods: A total of 39 patients with a history of COVID-19 6 months prior to the study were included in the study (Group I). Individuals were hospitalized or treated in the outpatients department. The control group (Group II) consisted of 39 healthy patients without a COVID-19 history. Each subject completed a questionnaire interview and underwent laboratory and pulmonary function examinations. Results: Six months after COVID-19 recovery, patients mainly complained about cough (46%, n = 18), shortness of breath (23%, n = 9), weakness (13%, n = 5), and memory/concentration disorders (8%, n = 3). In the group of patients complaining of persistent cough present 6 months after COVID-19, the following PFT parameters were decreased: FEV1, FVC, FRC, TLC, and DLCO (*p* < 0.05) in comparison with patients without this symptom. Conclusions: Persistent shortness of breath is not necessarily associated with pulmonary function impairment in patients 6 months after SARS-CoV-2 infection, and hence it requires appropriate differential diagnosis. Patients with a cough persisting 6 months after the acute phase of COVID-19 may benefit from PFT.

## 1. Introduction

Severe acute respiratory syndrome coronavirus 2 (SARS-CoV-2) causes coronavirus disease 2019 (COVID-19). It is a highly contagious virus that can be easily transmitted from human to human [1]. The COVID-19 pandemic has spread to all countries around the world. As of 31 October 2022, 627,104,342 confirmed cases of COVID-19, including 6,567,552 deaths, were reported to the WHO [2]. The clinical course of the disease varies. Symptoms of COVID-19 include cough, shortness of breath, fever, chest pain, headache, general malaise, myalgia, and taste or smell disturbance. In most cases, COVID-19 is mildly symptomatic. No hospitalization is required, and self-recovery occurs after several days. However, there is the possibility of an unfavorable evolution of the disease that would require hospitalization or eventually intensive setting, and the risk of death is 10%. This is mostly reported in elderly people, with concomitant illness (e.g., obesity, diabetes, hypertension, cardiovascular disease, chronic obstructive pulmonary disease) [3,4,5,6]. The impairment of ventilation and oxygenation depends on the degree of lung damage during the acute phase of COVID-19. The course of recovery from COVID-19 can have two unpredictable scenarios. First, with viral clearance and resolution of changes in the lungs, it can lead to complete recovery, and the second scenario, which is not fully understood, probably due to different fibrogenic molecular pathways that result in pulmonary injury, which may lead to the development of chronic respiratory failure and lung fibrosis [7,8]. On the basis of the studies conducted so far and clinical practice, we have already detailed information on pathogenesis, diagnosis, clinical symptoms, and therapeutic options in the acute phase of SARS-CoV-2 infection, as well as possible early complications [9,10]. However, we do not have precise knowledge about the long-term consequences of the disease and its impact on lung function [11,12,13,14]. Pulmonary function tests, including spirometry, lung volumes, and diffusing capacity of the lung for carbon monoxide, as well as respiratory muscle strength, airway resistance, or chest imaging, can help to determine the pulmonary consequences of COVID-19 disease objectively [15]. The growing number of COVID-19 convalescents requires a precise diagnostic tool and clear guidelines for clinicians on how to proceed in their daily practice [11,15]. From a clinical point of view, it is important to establish when, for whom, and how to monitor after SARS-CoV-2 infection [15]. The COVID-19 pandemic affects the population’s general health worldwide. Therefore, we should consider which diagnostic tests will reveal the ultimate health effect of SARS-CoV-2 infection in population medicine [16].

Our study aimed to investigate the impact of COVID-19 on the pulmonary function tests performed six months after recovery and answer whether these studies should be performed routinely in post-COVID-19 patients.

## 2. Materials and Methods

The study was conducted between 2 November 2020 and 12 February 2021, after the second wave of the COVID-19 pandemic in Poland.

### 2.1. Patient Selection

A total of 39 patients (26 women and 13 men) with a history of COVID-19 disease 6 months prior to the study were included in the study—Group I. The mean age was 47.0 ± 13.99 years (range: 26 to 78 years). The mean body mass index (BMI) in this group was 28.61 ± 5.51 kg/m^2^. The history of COVID-19 was established on the basis of the results of the reverse-transcription polymerase chain reaction (RT-PCR) test from nasopharyngeal swabs reported by the patient during a telephone interview and verified in the national SARS-CoV-2 database.

The control group (Group II) consisted of 39 healthy patients (26 women and 13 men) without a history of SARS-CoV-2 infection. The mean age in this group was 48.79 ± 13.88 years (range: 27 to 77 years). The mean body mass index was 28.60 ± 4.95 kg/m^2^.

Individuals were selected from the population study cohort—the Bialystok PLUS Study. Bialystok is a city in Poland with a population of almost 300,000 inhabitants. The Bialystok PLUS study describes the health of the Northeast Poland population of adults on the basis of a representative sample of the population of Bialystok city inhabitants. The Bialystok PLUS study not only concerns the current health status of the population providing valuable information about the development of diseases but also investigates psychological and sociological backgrounds that may affect them [17]. We randomly sampled individuals to Group II, in such a number that allowed us to obtain a similar age, gender, and body mass index. The study was conducted by the Medical University of Bialystok, Poland.

All included individuals were Caucasians, Bialystok inhabitants, and not yet vaccinated against COVID-19. The patients agreed to participate in the research and signed informed consent.

At the moment of the study, baseline oxygen saturation levels on atmospheric air were the same across these two groups (≥96%).

Each subject completed a questionnaire interview. Laboratory tests and pulmonary function tests were performed next.

On the basis of the treatment during the acute phase (at home or in hospital) and persistent symptoms six months after SARS-CoV-2 infection, patients from Group I were divided into the following subgroups: I a—with cough (n = 18), I b—without cough (n = 21), I c—with shortness of breath (n = 9), I d—without shortness of breath (n = 30), I e—treated in hospital (n = 16), I f—treated at home (n = 23). None of the hospitalized patients required mechanical ventilation or Intensive Care Unit treatment.

The study was conducted according to the guidelines of the Declaration of Helsinki and approved by the Bioethical Committee of the Medical University of Bialystok (Poland), protocol code: APK.002.346.2020.

### 2.2. Questionnaire

The questionnaire interview included health assessment at the moment of the study and before the pandemic, addictions, respondents’ and their family’s history of COVID-19 (clinical symptoms, the severity of the infection, treatment, hospitalization, persistent symptoms), the pandemic’s impact on their jobs, mental health, habits, medical history, and concomitant diseases. The questionnaire was completed with the interviewer using the LimeSurvey system.

### 2.3. Laboratory Test

Venous blood samples for laboratory tests were collected from all patients. The following laboratory blood parameters were measured according to a standardized procedure: white blood cell count (WBC), neutrophil count, lymphocyte count, monocyte count, red blood cell count (RBC), hemoglobin (HGB), and creatinine.

### 2.4. Pulmonary Function Test (PFT)

Pulmonary function tests were performed at BodyBox 5500 (Medisoft, Belgium) according to the American Thoracic Society and the European Respiratory Society guidelines [18].

Each subject underwent a full pulmonary function test consisting of spirometry, lung volumes, and diffusing capacity. Recorded parameters included forced vital capacity (FVC), forced expiratory volume in the first second (FEV1), FEV1/FVC ratio, functional residual capacity (FRC), total lung capacity (TLC), residual volume (RV), RV/TLC ratio, and diffusing capacity of the lung for carbon monoxide (DLCO, corrected for hemoglobin). The PFTs were performed on patients at the same time of day (before noon) by the same technician in all subjects.

The spirometry, lung volumes, and DLCO were expressed as median, extreme in liters, and a number of results lower than 80% of predicted normal values, respectively.

Staff performing PFT wore personal protective equipment (N95 face masks, eye goggles or face shields, gloves, and gowns). Disposable virus and bacterial filters were used during each test. Strict hand hygiene protocols were followed by the operator and the patient.

### 2.5. Statistical Analysis

Statistical analysis of collected data was performed using Statistica 13.0 software (StatSoft). The differences between the studied groups were assessed using the Mann–Whitney U test and the chi-squared test. Correlations between parameters were measured with Spearman’s rank correlation coefficient. *p*-values <0.05 were considered statistically significant.

## 3. Results

### 3.1. General Results

Demographic and clinical characteristics, the median, and extreme and statistical comparison of patients from Groups I and II are shown in Table 1.

Patients from Group I, during the acute phase of COVID-19, suffered from fever (92%, n = 36), dry (64%, n = 25) or productive (20%, n = 8) cough, weakness (95%, n = 37), shortness of breath (69%, n = 27), taste and smell disorder (79%, n = 31), palpitations (13%, n = 5), disturbances of consciousness (5%, n = 2), diarrhea (31%, n = 12), and skin rash (5%, n = 2). No thromboembolic complications were reported. Sixteen participants (41%) were admitted to the hospital. The median duration of hospitalization was 16 days (range of 4–30 days).

**Figure 1:**
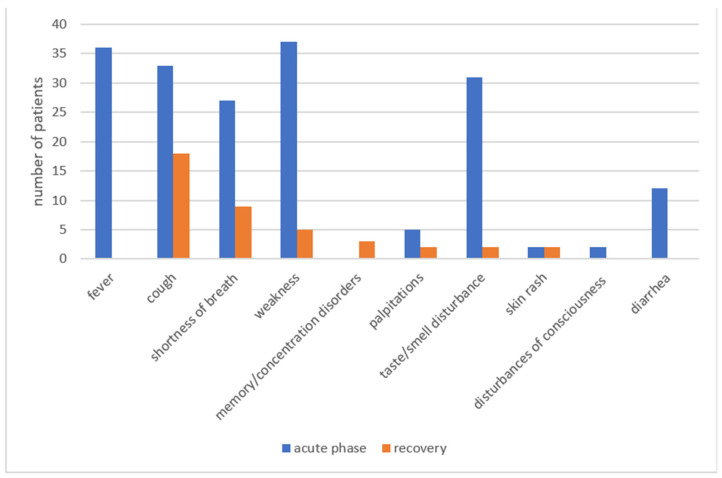
Group I—symptoms in the acute phase and 6 months after COVID-19.

Six months after recovery from COVID-19, eighteen patients (46%) still complained about cough, nine (23%) about the shortness of breath, five (13%) about weakness, and three (8%) about memory and concentration disorders. The following symptoms: palpitations, persistent taste/smell disturbance, and skin rash were reported equally by two patients (5%) each (Figure 1).

Fourteen patients (36%) had a history of smoking. One patient (3%) was an active smoker.

**Figure 2:**
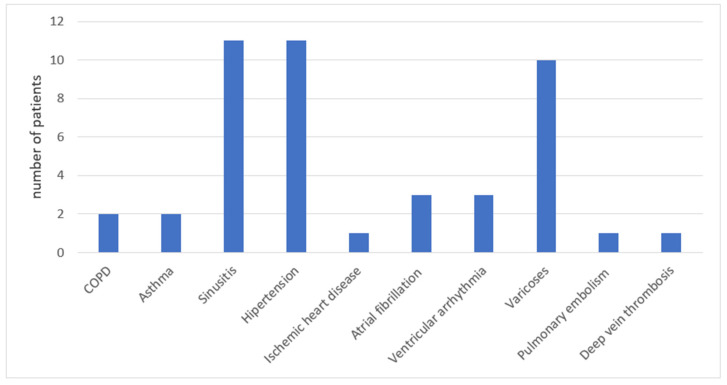
Group I—concomitant diseases.

Two (5%) of these individuals had previously been diagnosed with chronic obstructive pulmonary disease (COPD), two (5%) with asthma, and eleven (28%) had sinusitis in the past. Eleven (28%) suffered from arterial hypertension, one (2.5%) from ischemic heart disease, three (8%) from atrial fibrillation, three (8%) had ventricular arrhythmia, one (2.5%) had a history of pulmonary embolism, and one (2.5%) had deep vein thrombosis of the lower extremities (Figure 2). According to the patient’s opinion, concomitant diseases were well controlled.

Six (15%) patients used angiotensin-converting enzyme inhibitors in the management of arterial hypertension, and two (5%) patients used beta2-adrenergic agonists for obstructive pulmonary disease. The treatment was well tolerated. No possible side effects related to the therapy were reported before COVID-19.

Patients in Group II in their medical history reported arterial hypertension (n = 15, 38%), atrial fibrillation (n = 2, 5%), ventricular arrhythmia (n = 1, 2.5%), COPD (n = 1, 2.5%), asthma (n = 1, 2.5%), and sinusitis (n = 16, 41%). Concomitant diseases were well-controlled at the time of enrollment to the study, without any symptoms in the subject’s opinion.

### 3.2. Laboratory and Pulmonary Function Test (PFT)

Six months after COVID-19, no significant differences were found in laboratory tests and PFT compared to the control group. The median, quartiles, and statistical significance of laboratory tests and pulmonary function tests in patients from Groups I and II are shown in Table 1.

There was also no statistically significant difference in PFT depending on the presence of comorbidities and the drugs used among patients with a COVID-19 history.

In Group I a, the following parameters of pulmonary function tests were decreased: FEV1, FVC, FRC, TLC, and DLCO (*p* < 0.05) in comparison with Group I b. FEV1/FVC < 70% was confirmed only in two patients in Group I a and one patient in Group I b. Decreased DLCO (<80% of predicted values, corrected for hemoglobin) was observed in sixteen patients in Group I a (*p* < 0.013) (Table 2).

In patients from Group I c, no significant abnormalities in PFT were observed (Table 2).

No significant association between hospitalization due to COVID-19 and the results of PFT performed 6 months after recovery was observed in this group of patients (Table 2).

A higher value of FEV1, FVC, and TLC in the male population was also confirmed. This was due to the higher predicted values for sex, height, and weight. Among the female population, FEV1/FVC ratio <70% was observed in three cases.

## 4. Discussion

Although around two years have passed since the beginning of the COVID-19 pandemic, we still do not have precise follow-up protocols and guidelines on how to deal with convalescents [19]. The enormous number of all cases makes precise monitoring a challenge for healthcare systems. Various national/international organizations or societies’ guidelines allow practicing physicians to adapt and shape the way to organize their outpatient services locally. However, significant differences between the selected patients or technological recommendations regarding, e.g., the room ventilation in which the tests are performed, remain confusing [20]. Therefore, it is crucial to optimize the decision of when, for whom, and how to monitor after COVID-19 [15].

COVID-19 pneumonia is the most common clinical manifestation of SARS-CoV-2 infection [3]. Symptoms and clinical course of COVID-19 interstitial pneumonia depend on lung involvement and respiratory failure. The severe course of COVID-19, especially when ventilation support is needed, increases the risk of serious long-term respiratory consequences [19].

Mo et al. confirmed that in discharged COVID-19 survivors, decreased DLCO is the most common lung function abnormality. They also confirmed that the more severe the course of pneumonia was, the lower TLC was found [21]. Similar findings were reported in Huang’s study of fifty-seven patients 30 days after discharge from the hospital. Impaired DLCO was detected in more than half of the COVID-19 patients [12]. In the group of severe patients, a higher incidence of DLCO impairment and more TLC decrease were observed compared with non-severe cases [12,22]. The results of a study conducted by Ye et al. showed that almost half of the patients had mild-to-moderate DLCO dysfunction 3 months after discharge from the hospital [23].

Arnold et al.’s study revealed the persistence of symptoms at 12 weeks after the onset of symptoms or hospitalization despite confirmed improvement in clinical and radiological parameters. The most frequent symptoms were shortness of breath and excessive fatigue [24]. Our results were consistent with their findings. Cough (46%), shortness of breath (23%), and weakness (13%) were mainly reported by our patients 24 weeks after recovery.

A recent large cohort study (1733 patients) revealed that also 6 months after acute infection, COVID-19 survivors had impaired DLCO (22–56% across different severity scales). The intensity of the disorders correlated with the severity of the clinical course, and this group of patients seems to be the main target for long-term follow-up [25].

The long-term respiratory consequences of severe COVID-19 12 months after hospital discharge are also reported [26,27,28]. Most of the COVID-19 survivors showed a gradual improvement in their PFTs 3 months, 6 months, and 12 months after acute infection [26]. However, the persistent reduction in gas transfer measured by DLCO was observed one year after hospitalization in more than half of critically ill patients [26]. These findings are consistent with other COVID-19 1-year follow-up studies [27,28]. Steinbeis et al. emphasized the relevance of initial disease severity and pulmonary function impairment. They confirmed that lung involvement and respiratory failure in the acute phase were associated with a reduction in diffusion capacity in follow-up. What is more, respiratory symptoms did not improve significantly for patients with initially mild disease [27]. Increasing evidence suggests that a unified pathway for the respiratory follow-up of patients with COVID-19 is required [28].

Our results also showed impaired DLCO in 41% of patients who reported persistent cough 6 months after symptoms onset (*p* < 0.013). The median DLCO value was 4.20 L (IQR 1–3: 3.86–4.39), compared to 4.54 L in the group without cough (IOR 1–3: 4.38–4.79) (Table 2).

Surprisingly, no abnormalities in PFT were observed among patients who complained about the persistent feeling of shortness of breath. We suspect these patients may have had something other than the respiratory background of the reported complaints (e.g., cardiological disorders). Therefore, they require further multidisciplinary diagnostics to determine the cause of dyspnea.

The presence of a post-bronchodilator FEV1/FVC ratio < 70% confirms persistent airflow limitation. It is a gold standard to establish the diagnosis of chronic pulmonary obstructive disease (COPD) according to the GOLD report [29]. In our study, no broncho-dilatator was used; however, the initial value of FEV1/FVC ratio < 70% was observed in three patients in both the COVID-19 group and the control group. This abnormality appears not only to be related to SARS-CoV-2 infection but also to a history of exposure to tobacco smoke. It requires further diagnostic tests and appropriate pharmacological therapy in the case of a confirmation of persistent post-bronchodilator air-flow limitation.

Our study found no significant differences in PFT depending on the history of hospitalization. We speculate that it was because of a small number of patients enrolled in the study with a mild or moderate course of COVID-19 among them, according to the severity of the clinical symptoms and respiratory failure (mild—asymptomatic or oligosymptomatic; moderate—symptomatic without signs of respiratory failure; severe—severe pneumonia with respiratory failure/pre-ARDS; critical—ARDS/multi-organ failure). However, our results are in line with the study conducted by Lewis et al. who compared pre-infection and post-infection PFT in 80 COVID-19 patients. No differences in PFT before and after SARS-CoV-2 infection in non-critically ill classified patients were observed [30]. Further research is needed to compare the long-term outcomes between inpatients and outpatients with various clinical courses.

The main limitation of our study was related to the low number of patients enrolled in both groups. Similar studies should be carried out on a much larger number of patients, including patients from the next waves of the COVID-19 pandemic, as much has changed since the beginning of 2020. Furthermore, another limitation of our study was the lack of access to hospitalization data because it was a pilot study based on a questionnaire and answers obtained from patients. We admit that data from a hospitalization would be of great value, but it was analysis from the beginning of the pandemic, and not all patients were hospitalized. Therefore, we believe that the results of this study should be treated as an introduction to further studies. However, we would like to emphasize that our study provided additional knowledge regarding the indications for a specific diagnostic tool in post-COVID-19 patients. From a clinical point of view, this will impact physicians dealing with COVID-19 survivors as the number of patients after the SARS-CoV-2 infection continues to increase. Even if COVID-19 remains endemic in the future, it will become a part of routine clinical practice.

## 5. Conclusions

PFTs as a screening investigation in patients with no symptoms from the respiratory tract 6 months after COVID-19 are not useful. Persistent shortness of breath is not necessarily associated with pulmonary function impairment in patients 6 months after SARS-CoV-2 infection, and hence it requires appropriate differential diagnosis. Patients with persistent cough present 6 months after the acute phase of COVID-19 may benefit from PFT.

## Figures and Tables

**Table 1:** Demographic and clinical characteristics, the median, and extreme and statistical significance of laboratory tests and pulmonary function tests in patients 6 months after COVID-19 (post-COVID-19) and the control group (CG).

Characteristic/Parameter	Group I (Post-COVID-19)	Group II (Control Group)	*p*
Age			1
Median	47.00	48.79	
Min.–max.	26.0–78.0	27.00–77.00	
Sex			0.92
Male	13	13	
Female	26	26	
BMI (kg/m^2^)			0.960
Median	28.61	28.60	
Min.–max.	20.22–40.52	20.22–40.52	
WBC count (cells/μL)			0.726
Normal range 4000–10,000/μL			
Median	5900	6100	
Min.–max.	3700–14,000	4700–11,900	
Lymphocyte (cells/μL)			0.681
Normal range 800–4700/μL			
Median	2000	1900	
Min.–max.	1200–3900	1200–4400	
Neutrophil (cells/μL)			0.663
Normal range 1600–7200/μL			
Median	3800	3800	
Min.–max.	1700–12,200	2600–10,000	
Monocyte (cells/μL)			0.046
Normal range 100–1000/μL			
Median	300	300	
Min.–max.	200–500	200–600	
RBC count (cells/μL)			0.069
Normal range 4,000,000–5,500,000/μL			
Median	4,500,000	4,710,000	
Min.–max.	3,860,000–5,320,000	4,030,000–5,750,000	
HGB (g/dL)			0.112
Normal range 12.00–16.00 g/dL			
Median	13.5	14.00	
Min.–max.	11.4–16.3	11.10–16.30	
CREA (mg/dL)			0.026
Normal range 0.73–1.18 mg/dL			
Median	0.65	0.72	
Min.–max.	0.51–1.20	0.50–1.07	
FEV1 (L)			0.584
Median	3.37	3.18	
Min.–max.	1.96–5.69	1.5–5.88	
<80% of predicted	n = 2	n = 2	
FVC (L)			0.861
Median	3.96	4.02	
Min.–max.	2.49–6.88	2.17–7.13	
<80% of predicted	n = 0	n = 0	
FEV1/FVC ratio (%)			0.487
Median	78.8	78.6	
Min.–max.	64.7–92.50	43.7–86.30	
<70%	n = 3	n = 3	
FRC (L)			0.065
Median	2.29	2.71	
Min.–max.	1.49–3.85	1.78–4.04	
TLC (L)			0.657
Median	5.38	5.8	
Min.–max.	3.99–8.92	3.67–9.05	
<80% of predicted	n = 1	n = 1	
RV (L)			0.121
Median	1.42	1.63	
Min.–max.	0.3–2.5	0.56–2.61	
RV/TLC radio (%)			0.352
Median	22.26	26.6	
Min.–max.	5.4–40.93	12.20–48.51	
DLCO (L)			0.351
Median	4.38	4.53	
Min.–max.	3.14–5.32	2.77–5.66	
<80% of predicted	n = 29	n = 25	

BMI = body mass index. WBC = white blood cell count. Lymphocyte = lymphocyte count. Neutrophil = neutrophil count. Monocyte = monocyte count. RBC = red blood cell count. HGB = hemoglobin. CREA = creatinine. FEV1 = forced expiratory volume in the first second. FVC = forced vital capacity. FRC = functional residual capacity. TLC = total lung capacity. RV = residual volume. DLCO = diffusing capacity of the lung for carbon monoxide (corrected for hemoglobin).

**Table 2:** The median, range, and statistical significance of laboratory tests and pulmonary function tests in patients 6 months after COVID-19 (post-COVID-19) who reported a persistent cough and shortness of breath, depending on hospitalization during the acute phase of infection.

Parameter	Cough			Shortness of Breath			Hospitalization		
	Yes (Group I a) (n = 18)	No (Group I b) (n = 21)	*p*	Yes (Group I c) (n = 9)	No (Group I d) (n = 30)	*p*	Yes (Group I e) (n = 16)	No (Group I f) (n = 23)	*p*
WBC (cells/μL) Normal range 4000–10,000/μL			0.651			0.483			0.303
Median	5650	6500		5900	5950		5800	6000	
IQR 1–3	5000–7500	4800–7800		5600–7800	4700–7500		4300–7200	5200–8000	
Lymphocyte (cells/μL) Normal range 800–4700/μL			0.252			0.132			0.002
Median	2000	2000		2500	1900		1600	2100	
IQR 1–3	1800–2500	1400–2300		1900–2500	1600–2300		1450–1850	1900–2500	
Neutrophil (cells/μL) Normal range 1600–7200/μL			0.337			0.776			0.775
Median	3300	4000		3400	3850		3700	3800	
IQR 1–3	2800–4400	2900–5100		3200–5100	2700–5200		2700–5100	2900–5100	
Monocyte (cells/μL) Normal range 100–1000/μL			0.243			0.269			0.107
Median	300	300		300	300		300	300	
IQR 1–3	200–400	200–300		300–400	200–300		200–300	300–300	
RBC (cells/μL) Normal range 4,000,000–5,500,000/μL			0.15			0.309			0.303
Median	4,400,000	4,550,000		4,530,000	4,480,000		4,435,000	4,580,000	
IQR 1–3	4,280,000–4,690,000	4,390,000–7,970,000		4,330,000–4,970,000	4,270,000–4,780,000		4,305,000–4,730,000	4,290,000–4,970,000	
HGB (g/dL) Normal range 12.00–16.00 g/dL			0.104			0.229			0.235
Median	13.2	13.9		14.3	13.4		13.4	13.9	
IQR 1–3	12.7–14.2	13.3–15.2		12.9–15.5	12.9–14.2		12.9–14.1	12.9–15.4	
FEV1 (L)			0.02			0.798			0.249
Median	2.85	3.82		3.18	3.46		3.11	3.54	
IQR 1–3	2.29–3.58	2.88–4.31		2.65–4.03	2.5–8.89		2.35–3.64	2.59–4.3	
<80% of pred.	n = 1	n = 1		n = 1	n = 1		n = 1	n = 1	
FVC (L)			0.021			0.77			0.199
Median	3.55	4.87		3.63	4.21		3.73	4.32	
IQR 1-3	3.15–4.34	3.55–5.58		3.43–5.12	3.15–5.04		3.12–4.67	3.55–5.47	
<80% of pred.	n = 0	n = 0		n = 0	n = 0		n = 0	n = 0	
FEV1/FVC ratio (%)			0.962			0.962			0.188
Median	78.7	78.9		78.8	78.8		78.7	79.25	
IQR 1–3	76.8–82.2	76.7–83.8		74.6–83.2	76.8–82.2		77.3–80.6	75.2–83.8	
<70%	n = 2	n = 1		n = 2	n = 1		n = 1	n = 2	
FRC (L)			0.031			0.756			0.299
Median	2.2	3.02		2.28	2.3		2.23	2.58	
IQR 1–3	1.87–2.45	2.28–3.33		2.1–3.01	1.92–3.26		1.87–2.5	2.18–3.23	
TLC (L)			0.038			0.609			0.322
Median	5.135	6.59		5.76	5.23		5.03	5.8	
IQR 1–3	4.82–5.6	4.78–7.59		5.32–5.91	4.75–7.19		4.78–5.85	4.86–7.19	
<80% of pred.	n = 1	n = 1		n = 1	n = 0		n = 0	n = 1	
RV (L)			0.824			0.442			0.896
Median	1.53	1.41		1.61	1.31		1.36	1.42	
IQR 1–3	0.91–1.72	1.18–1.98		1.4–1.72	0.91–1.83		0.99–1.66	0.89–1.72	
RV/TLC radio (%)			0.35			0.609			0.338
Median	29.27	21.69		29.49	22.21		29.32	22.07	
IQR 1–3	18.46–33.76	18.56–29.6		20.74–31.14	18.46–29.99		19.53–31.63	18.19–29.87	
DLCO (L)			0.013			0.661			0.372
Median	4.2	4.54		4.45	4.37		4.35	4.42	
IQR 1–3	3.86–4.39	4.38–4.79		4.2–4.66	3.88–4.71		3.8–4.68	4.06–4.71	
<80% of pred.	n = 16	n = 13		n = 6	n = 23		n = 13	n = 16	

BMI = body mass index. WBC = white blood cell count. Lymphocyte = lymphocyte count. Neutrophil = neutrophil count. Monocyte = monocyte count. RBC = red blood cell count. HGB = hemoglobin. FEV1 = forced expiratory volume in the first second. FVC = forced vital capacity. FRC = functional residual capacity. TLC = total lung capacity. RV = residual volume. DLCO = diffusing capacity of the lung for carbon monoxide (corrected for hemoglobin).

## Data Availability

The datasets used and/or analyzed during the current study are available from the corresponding author upon reasonable request.
